# Use of Insulin to Increase Epiblast Cell Number: Towards a New Approach for Improving ESC Isolation from Human Embryos

**DOI:** 10.1155/2013/150901

**Published:** 2013-02-12

**Authors:** Jared M. Campbell, Michelle Lane, Ivan Vassiliev, Mark B. Nottle

**Affiliations:** ^1^Discipline of Obstetrics and Gynaecology, School of Paediatrics and Reproductive Health, University of Adelaide, Medical School South, Level 3, Frome Road, Adelaide, SA 5005, Australia; ^2^Centre for Stem Cell Research, University of Adelaide, Medical School South, Level 3, Frome Road, Adelaide, SA 5005, Australia; ^3^Repromed, 180 Fullarton Road, Dulwich, SA 5065, Australia

## Abstract

Human embryos donated for embryonic stem cell (ESC) derivation have often been cryopreserved for 5–10 years. As a consequence, many of these embryos have been cultured in media now known to affect embryo viability and the number of ESC progenitor epiblast cells. Historically, these conditions supported only low levels of blastocyst development necessitating their transfer or cryopreservation at the 4–8-cell stage. As such, these embryos are donated at the cleavage stage and require further culture to the blastocyst stage before hESC derivation can be attempted. These are generally of poor quality, and, consequently, the efficiency of hESC derivation is low. Recent work using a mouse model has shown that the culture of embryos from the cleavage stage with insulin to day 6 increases the blastocyst epiblast cell number, which in turn increases the number of pluripotent cells in outgrowths following plating, and results in an increased capacity to give rise to ESCs. These findings suggest that culture with insulin may provide a strategy to improve the efficiency with which hESCs are derived from embryos donated at the cleavage stage.

## 1. Introduction

The embryo begins as a single totipotent cell it then undergoes multiple rounds of division coupled with differentiation until it forms a blastocyst and has the potential to implant in the uterus. The pluripotent epiblast of the inner cell mass (ICM) then undergoes further division and differentiation to develop into the fetus and eventually a fully developed organism. Epiblast cells can be isolated and cultured in conditions which allow embryonic stem cell (ESC) lines to be derived. ESC lines, especially hESC lines, hold considerable promise in the fields of drug discovery, developmental biology and regenerative medicine. However, the efficiency of hESC derivation is low. This paper brings together work by our group which used a mouse model to develop a strategy for improving the efficiency of hESC derivation. As most embryos donated for human ESC derivation were cultured in relatively simple media, now known to perturb development, before being frozen at the precompaction stage up to 10 years earlier, we examined the hypothesis that the period where they are subsequently cultured to the blastocyst stage could be exploited to improve the efficiency with which cell lines could be derived. In particular, our work has focussed on adding insulin to culture media to increase epiblast cell number. 

In initial studies [1], we showed that in vitro culture of embryos during the precompaction stage in a simple medium that was designed to model the culture conditions that human embryos available for hESC derivation were previously exposed to reduces embryo quality; as highlighted by reduced developmental rates, decreased epiblast cell number and altered gene expression in outgrowths compared with that seen for embryos culture in modern G1 medium. Some aspects of this reduction in quality could be restored after compaction by culture in a medium designed to support postcompaction embryo development in vitro (G2), including epiblast cell number. However, epiblast cell number was only partially improved compared with embryos cultured in G1/G2. The findings of [2, 3] as well as our own later studies [4] suggest that increased epiblast cell number correlates with an increased capacity to give rise to ESCs. Culture in G2 medium postcompaction also increased the proportion of embryos which reached the hatched blastocyst stage which was subsequently shown in [4] to be correlated with an increased capacity to give rise to primary ESC colonies. Together, these findings highlight that subsequent culture in modern culture systems can improve the efficiency of ESC derivation from embryos initially cultured in simple media.

Numerous growth factors are known to influence embryo development. However, these are not routinely included in embryo culture media. In contrast, growth factors such as leukaemia inhibitory factor (LIF) are routinely used for the isolation and maintenance of ESCs. Based on these observations, we hypothesised that addition of a growth factor to embryo culture media could further improve blastocyst development, epiblast cell number, outgrowth formation rate, and ESC derivation. In particular, we investigated whether insulin could be used to increase ESC derivation efficiency because it has previously been shown to increase ICM cell number when added to embryo culture media [5, 6].

The results from these studies showed that the inclusion of insulin in postcompaction culture medium increased the number of pluripotent cells in blastocysts. In a series of experiments we showed that insulin acted via the PI3K/GSK3 p53 pathway to shift the balance of differentiation versus pluripotency within the ICM to increase epiblast number and proportion [7]. This resulted in an increase in their capacity to give rise to outgrowths with more pluripotent cells, as well as an increase in capacity to give rise to primary ESC colonies [4]. These findings suggest that the inclusion of insulin in embryo culture medium postcompaction could be used to improve the quality of cryopreserved or fresh human embryos donated at or near compaction. 

## 2. Impact of Culture Conditions on Epiblast Cell Number and Pluripotency

The relatively low efficiency with which hESCs can be derived has been attributed to the reduced quality of human embryos donated for this purpose [8–10]. In vitro culture of embryos is typically associated with reductions in embryo quality and viability [11]. Furthermore, human embryos for hESC derivation have often been cryopreserved for 5–10 years prior to their donation [12, 13]. As a consequence many were cultured in media now known to perturb viability, and which supported only low levels of blastocyst development. This necessitated cleavage stage transfers for the majority of IVF cycles performed. 

In order to model this system, mouse embryos were cultured in relatively simple medium for the first 48 h to the 8-cell stage to approximate the culture period commonly used in human IVF experiments [1]. These experiments demonstrated that culture of embryos in simple medium retarded the development of blastocysts and significantly reduced the number of epiblast cells, which was consistent with previous studies in human and mouse [14–19]. These experiments also demonstrated that there was some capacity to improve blastocyst development and epiblast cell number by transferring embryos to the more complex G2 medium for culture from the 8-cell stage. Despite this, initial culture in relatively simple medium had a lasting negative impact on subsequent development. Furthermore, we found that embryos cultured in simple medium were less likely to contain an epiblast and therefore lacked the capacity to generate an ESC line, irrespective of the culture medium used for the second 48 h. Additionally, assessment of outgrowths generated from these blastocysts showed that the perturbing conditions of a simple medium had lasting effects on the gene expression of the outgrowths, with altered gene expression of *Atrx* and *Nanog*.

Human embryos donated for hESC derivation are likely to have been exposed to conditions such as simple style culture medium, examples of which include HTF, Earle's, and T6, which were widely used in IVF and can still be found in use. Collectively, the findings of [1] therefore indicate that these embryos are likely to have a reduced capacity to give rise to hESCs. This in turn suggests that the characteristics of hESC lines could be affected by predonation embryo culture conditions.

As many human embryos, historically and presently, are cryopreserved at the cleavage stage [20–22] and therefore donated for ESC generation at this stage, they must be further cultured to the blastocyst stage before ESC derivation. This additional culture period represents a window where the pluripotency of embryos which have previously been exposed to perturbing culture conditions can be improved. The results of [1] demonstrated that while the quality of mouse embryos could be improved by culturing them in modern complex medium purpose designed to support embryo development from the 8-cell stage, additional interventions are necessary to fully exploit the cleavage to blastocyst culture period. 

## 3. Insulin Stimulation of Pluripotency in Postcompaction Embryos

The inclusion of select growth factors in embryo culture media has previously been shown to be capable of improving embryo development and viability [5, 23]. However, growth factors are not routinely included in culture media commercially available for human embryo culture [24–28]. To further examine how interventions to the culture medium for the postcompaction stage embryo may affect epiblast cells and pluripotency, the growth factor insulin was added to the culture medium. Insulin has previously been shown to increase the ICM cell number of embryos [29] and was selected as the most promising candidate from a panel of growth factors previously used to improve embryo culture. 

The findings of [7] demonstrated that 1.7*ρ*M insulin increased epiblast cell number without affecting ICM cell number. This resulted in a significant increase in the proportion of ICM cells which were epiblast as opposed to primitive endoderm. This novel finding suggested that insulin was acting to shift the balance of differentiation within the ICM towards more pluripotent cells, rather than acting as a general mitogenic factor and stimulating overall cell growth. This was further highlighted by the finding that total cell number and trophectoderm cell number were also unaffected by the addition of insulin. However, as in previous studies [5, 23], there was a threshold concentration where the effect of insulin was maximal, above which further increases led to the loss of the increase in epiblast cell number. If this strategy is implemented in the human, it may be necessary to repeat these dose response experiments to establish an optimal dose in terms of epiblast cell number increases. While this work shows that insulin is able to maintain pluripotency in the ICM and direct differentiation, other growth factors may also have beneficial effects, and it is possible that a combination of growth factors may produce a synergistic effect and improve blastocyst quality and epiblast cell number.

## 4. Molecular Mechanism of Action of Insulin on Pluripotency in the Blastocyst

**Figure 1:**
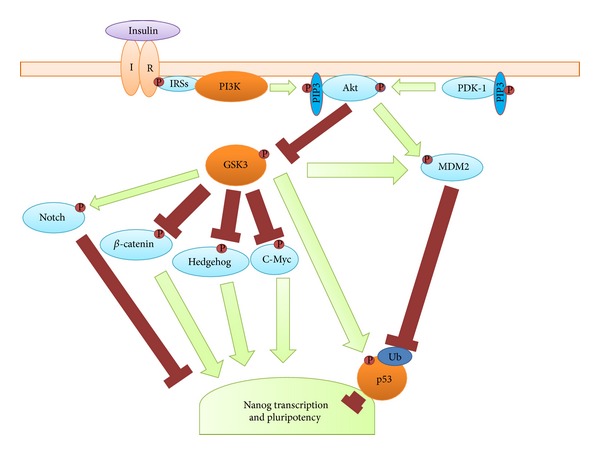
Schematic of insulin signalling and its regulation of Nanog expression and pluripotency. Green arrows indicate reactions with a stimulatory effect on their target, and red closed bars indicate reactions with a retarding effect on their target. P marks reactions where phosphorylation occurs, and Ub marks reactions where ubiquitination occurs. Insulin binds the insulin receptor (IR), a tyrosine kinase which is then able to phosphorylate the IRSs. PI3K is able to bind to the phosphorylated IRSs by its SH2 domains, resulting in activation. PI3K phosphorylates the phospholipid PIP2, producing PIP3, which can be bound by the pleckstrin homology domains of PDK-1 and Akt. Results in [7] show that activation of PI3K is necessary for insulin to increase the number of Nanog positive epiblast cells during embryo culture. When PDK-1 and Akt are colocalised to the cell membrane, PDK-1 is able to phosphorylate and activate Akt. Active Akt can phosphorylate GSK3, inactivating it. When active, GSK3 is able to phosphorylate *β*-catenin, Hedgehog, and c-Myc; all factors which safeguard pluripotency through interactions with other second messengers. Additionally, active GSK3 phosphorylates and protects the intracellular domain of Notch, promoting differentiation. Further, inactivation of GSK3 is necessary for insulin to increase the number of Nanog positive epiblast cells during embryo culture [7]. Akt is also able to phosphorylate and activate MDM2 which ubiquitinates the proapoptotic factor p53, causing its inactivation and removal from the nucleus, where it would bind to the *Nanog* promoter and suppresses its expression. Inactivation of p53 is necessary for insulin to increase the number of Nanog positive epiblast cells during embryo culture [7]. GSK3 and p53 are able to form a dimer, resulting in the phosphorylation of p53 and the increased activity of both factors. GSK3 is also able to phosphorylate and activate MDM2. However, despite these outcomes, the interaction of GSK3 and p53 do not have a significant effect on Nanog positive epiblast cell number during embryo culture [7].

Having demonstrated that the culture of postcompaction stage embryos with insulin increases epiblast cell number, further experiments were undertaken to determine the signalling pathways behind this effect [7]. At the concentration identified as increasing epiblast cell number and proportion, insulin is known to activate the insulin receptor [29]. One of the primary second messengers of the insulin receptor is PI3K, which has previously been shown to be integral for maintaining pluripotency in ESCs [30]. Using inhibitors, it was demonstrated that PI3K activity was necessary for insulin to increase epiblast cell number (Figure 1). One target of PI3K is GSK3, which is phosphorylated by active PI3K, inactivating it. When active, GSK3 is capable of phosphorylating many second messengers which converge to reduce Nanog transcription, which is important for the retention of pluripotency [31–37]. Inhibiting GSK3, theoretically reproducing the effect of culture with insulin and active PI3K, increased epiblast cell number. Furthermore, activating GSK3 blocked insulin's ability to increase epiblast cell number without affecting the epiblast cell number of embryos cultured without insulin, replicating the effect of PI3K inhibition. These results suggest that the inactivation of GSK3 is an important component of the insulin signalling pathway in relation to increasing epiblast cell number. 

The pro-apoptotic protein p53, which is also regulated by PI3K, specifically via PI3K activated ubiquitinase MDM2 [38–40], causes cell death and differentiation when active and binds to the *Nanog* promoter region to repress Nanog transcription [41]. As with GSK3, inhibition of p53 increased epiblast cell number, while activation blocked insulin-mediated epiblast increases, strongly suggesting that p53 is involved in insulin-mediated increases to epiblast cell number. Interestingly, there are multiple points of cross-reactivity between GSK3 and p53 [42–46]; however, no additional epiblast increases were found for co-inhibition of the two factors. This suggests that the potential of GSK3 inhibition to cause the accumulation of p53 [45, 46] did not have a confounding effect in these experiments. In conclusion, the results of these studies demonstrated that insulin increased the epiblast cell number via the activation of PI3K (most likely via its interaction with the insulin receptor), which subsequently inactivates the second messengers GSK3 and p53, to increase Nanog transcription and therefore promote pluripotency and the epiblast (Figure 1).

## 5. Expression of OCT4 and Nanog in Blastocysts

The localisation of OCT4 and Nanog in blastocysts on day 4 (early blastocysts), day 5 (predominantly expanded blastocyst), and day 6 (hatching blastocysts) was determined by immunohistochemistry [4]. Of note, it was found that at the stage of development where the literature sources suggested that OCT4 and Nanog would be restricted to the ICM and epiblast, respectively [47, 48], both were still widely expressed. A comparison of methodologies suggested that this difference is likely the result of collecting and beginning embryo culture at the zygote stage in this study, rather than at the 2-cell stage or later. 

Human embryos are ubiquitously cultured from the zygote stage following in vitro fertilisation. As such, this finding suggests that future researchers who use the mouse blastocyst to model the in vitro development of human embryos should culture embryos from the zygote stage, as the discrepancy appears to produce a meaningful difference particularly with regards to epiblast development. Future work in this area should include the direct comparison of OCT4 and Nanog expression of in vitro and in vivo grown mouse embryos as well as the characterisation of Oct4 and Nanog expression in human blastocysts.

## 6. Effect of Insulin in Embryo Culture Medium Persists in Outgrowths

Despite the increase in Nanog positive cell number due to culture with insulin, we found that when blastocysts were plated before the transcription factor was restricted to the epiblast, outgrowths from insulin-treated embryos contained no more epiblast cells than outgrowths from control embryos. Further, our results showed that despite earlier stage blastocysts possessing more OCT4 and Nanog positive cells than later stage blastocysts, they gave rise to outgrowths with significantly fewer epiblast cells. As hESCs have been shown to be most efficiently derived from blastocyst where Oct4 has been restricted to the ICM [49], this finding supports the use of mouse blastocysts in modelling human embryo development and hESC derivation. The important finding from these experiments, however, was that when embryos were allowed to develop until Nanog was restricted to the epiblast and OCT4 was restricted to the ICM before plating, culture of embryos in insulin postcompaction resulted in the generation of outgrowths which were more likely to contain an epiblast and which contained a larger number of epiblast cells. 

## 7. Insulin in Culture Media and the Effect of ESC Colony Generation

Insulin- and control-treated blastocysts were plated on day 6, outgrown, trypsinised, and replated, and primary cell colonies with an ESC morphology were stained for OCT4 and Nanog expression to confirm pluripotency [4]. Blastocysts were twice as likely to give rise to primary ESC colonies if they were cultured with insulin for the postcompaction stage. Interestingly, examining how the inclusion of insulin interacted with this process led to the novel observation that culture of embryos with insulin increased the proportion of embryos which, at the point of plating, were at the most advanced morphological stage (hatched) and also increased the proportion of those hatched blastocysts which gave rise to ESCs. As such, hatched insulin-cultured blastocysts are more plentiful and more likely to give rise to ESCs than hatched control-cultured blastocysts. This result demonstrates that insulin improves ESC isolation through mechanisms beyond simply improving morphology, which has previously been linked to increased ESC derivation rates. It is likely that the improved capacity of insulin-cultured blastocysts with the highest morphological quality to give rise to primary ESC colonies is the result of the increased epiblast cell numbers demonstrated in both [4, 7].

Modelling of the experimental outcomes enabled conclusions to be made around the most significant characteristics that an embryo must contain to generate a primary ESC colony. The greatest predictor of a control-cultured blastocyst giving rise to a primary ESC colony was it cavitating on day 4, whereas for blastocysts cultured with insulin, the greatest predictor was being hatched on day 6. For day 4, this observation is likely the result of insulin increasing the rate of cavitation and thereby making the marker less selective. The finding on day 6 is suggestive that in the control group, hatched blastocysts, which have shown the best development, have no more epiblast cells than their more slowly developing counterparts. Both of these observations warrant further investigation.

The results of this work demonstrate that the addition of insulin to embryo culture medium from the cleavage stage to the blastocyst stage improves the efficiency with which ESCs can be generated from these embryos. As human embryos are most often donated at the cleavage stage and ESC derivation is most often attempted at the blastocyst stage, the application of this strategy has the potential to improve hESC derivation efficiency. Due to the limited availability of human embryos for ESC derivation, improving efficiency is a matter of key importance. 

Future work which would be necessary to validate these findings is the expansion of mESC colonies from control- and insulin-cultured embryos to fully characterised mESC lines and the reproduction of these experiments in the human. Further, our work has shown that the effect of insulin persists beyond embryo culture through the outgrowth phase and into ESC derivation. This suggests that during embryo culture-insulin may have a permanent positive effect on cell properties and that ESC lines derived from embryos cultured with insulin may have altered characteristics. As such, future work should include not just the characterisation of ESC lines from control and insulin-cultured embryos for pluripotency and self-renewal, but also more in depth characterisation including metabolic profile, an assessment of DNA methylation and acetylation, and gene expression, to provide a more detailed and precise picture of the quality and differentiation status of ESC lines, with a view towards investigating whether the culture of embryos with insulin results in the derivation of higher quality ESC lines.

## 8. Conclusion

**Figure 2:**
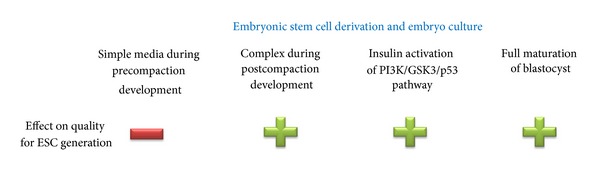
Summary of the culture effects examined in this paper and their observed effect on the retention of pluripotency towards ESC derivation.

In conclusion, the results presented in this paper show that while culture in simple medium during the cleavage stage decreases pluripotency, the inclusion of insulin in embryo culture medium from the compaction stage stimulates pluripotency supporting pathways to increase the number of epiblast cells in the fully developed blastocyst, resulting in an increased capacity to generate ESCs (Figure 2). This strategy is of particular relevance for hESC derivation where embryos are most often donated at the cleavage stage and of reduced quality.
